# Large scale genotyping study for asthma in the Japanese population

**DOI:** 10.1186/1756-0500-2-54

**Published:** 2009-03-31

**Authors:** Yoshiko Imada, Masaya Fujimoto, Kenji Hirata, Tomomitsu Hirota, Yoichi Suzuki, Hirohisa Saito, Kenji Matsumoto, Akira Akazawa, Toshio Katsunuma, Shigemi Yoshihara, Motohiro Ebisawa, Masanao Shibasaki, Tadao Arinami, Mayumi Tamari, Emiko Noguchi

**Affiliations:** 1 Department of Medical Genetics, Graduate School of Comprehensive Human Sciences, University of Tsukuba, Tsukuba, 305-8577 Japan; 2 Laboratory of Genetics of Allergic Diseases, RIKEN SNP Research Center, Yokohama, Japan; 3 Department of Public Health Chiba Graduate University School of Medicine, Chiba, Japan; 4 National Research Institute for Child Health & Development, Tokyo, Japan; 5 Department of Pediatrics, Tokyo Jikeikai school of Medicine, Tokyo, Japan; 6 Dokkyo Medical University, Tochigi, Japan; 7 Clinical Research Center for Allergy, National Sagamihara Hospital, Kanagawa, Japan; 8 Department of Pediatrics, Tsukuba College of Technology, Tsukuba, Japan

## Abstract

**Background:**

Asthma is a complex phenotype that is influenced by both genetic and environmental factors. Genome-wide linkage and association studies have been performed to identify susceptibility genes for asthma. These studies identified new genes and pathways implicated in this disease, many of which were previously unknown.

**Objective:**

To perform a large-scale genotyping study to identify asthma-susceptibility genes in the Japanese population.

**Methods:**

We performed a large-scale, three-stage association study on 288 atopic asthmatics and 1032 controls, by using multiplex PCR-Invader assay methods at 82,935 single nucleotide polymorphisms (SNPs) (1^st ^stage). SNPs that were strongly associated with asthma were further genotyped in samples from asthmatic families (216 families, 762 members, 2^nd ^stage), 541 independent patients, and 744 controls (3^rd ^stage).

**Results:**

SNPs located in the 5' region of *PEX19 *(rs2820421) were significantly associated with *P *< 0.05 through the 1^st ^to the 3^rd ^stage analyses; however, the *P *values did not reach statistically significant levels (combined, *P *= 3.8 × 10^-5^; statistically significant levels with Bonferroni correction, *P *= 6.57 × 10^-7^). SNPs on *HPCAL1 *(rs3771140) and on *IL18R1 *(rs3213733) were associated with asthma in the 1^st ^and 2^nd ^stage analyses, but the associations were not observed in the 3^rd ^stage analysis.

**Conclusion:**

No association attained genome-wide significance, but several loci for possible association emerged. Future studies are required to validate these results for the prevention and treatment of asthma.

## Findings

Asthma is the most common chronic disorder in children, and asthma exacerbation is an important cause of childhood morbidity and hospitalization. Currently, approximately 300 million people worldwide have asthma, and this disease claims the lives of 180,000 people every year [1].

Asthma and atopy are complex phenotypes that are influenced by both genetic and environmental factors. Twin studies have supported the role of a strong genetic contribution with a heritability of 0.71 [2], and asthma shows a familial risk higher than that of many common diseases such as stroke, epilepsy, and most types of cancer [3]. Atopy is characterized by increased levels of immunoglobulin E (IgE) against common environmental allergens, and is considered the strongest predisposing factor for asthma. Majority of children with asthma develop specific IgE against house dust mites, and dust mite allergy is strongly associated with asthma [4,5], however, only a small subset of subjects with dust mite allergy develops asthma [6]. These data suggest that other factors are involved in the development of asthma, and genome-wide linkage and association studies have been used to find novel asthma genes and their associated pathways [7]. Using family and case-referent panels of European populations and based on the genome-wide association study conducted on asthma, Moffatt *et al*. identified that the cis-acting single nucleotide polymorphisms (SNPs) in *ORMDL3 *were associated with asthma [8], and the results were replicated in independent populations [9].

In order to identify novel asthma susceptibility genes, we performed a large-scale, 3-stage association study using the Japanese population. No association attained genome-wide significance, but several loci for possible association emerged. Further studies are required to validate these results in the future.

### Subjects and genotyping results

The childhood asthmatics in the case-control study (1^st ^and 3^rd ^stage analysis) were atopic asthmatic children diagnosed by pediatricians on the basis of clinical examination. Probands of the asthmatic families (2^nd ^stage) were atopic asthmatic children who visited the Pediatric Allergy Clinic of the University Hospital of Tsukuba. Two hundred and sixteen families (762 members), provided informed consent and participated in this study. The clinical details of the families are shown in Table 1. The criteria used for the diagnosis of asthma in case-control study and families were the same, and have been previously described [10]. The control group comprised 1032 adult Japanese individuals from the general population (1^st ^stage) and 744 healthy adults (ages, 19–78 years, mean 46.2 years) with no history of any allergic disease (3^rd ^stage). Cases and controls as well as asthmatic families were recruited from the mainland of Japan. This study was approved by the Committee of Ethics of the University of Tsukuba. The details of the study populations are shown in Table 2.

**Table 1:** Clinical details of the asthmatic families

No. of Families	216
No. of children	346
No. of affected children	315
Mean age (yeas ± SD)	10.9 ± 2.4
Male:Female ratio	1.8:1
Log(total IgE) (IU/ml ± SD)	2.8 ± 0.6
No. of parents	416
Mean age (yeas ± SD)	40.7 ± 7.5
Log(total IgE) (IU/ml ± SD)	2.0 ± 0.7

**Table 2:** Study design

	Case	Control	Analysis	No of SNPs
1st stage	Childhood atopic Asthma: 288	General Japanese population: 1032	χ^2 ^test (allelic)	82,935
2nd stage	Family with childhood atopic asthma (216 families, 762 members)		PDT	125
3rd stage	Childhood atopic Asthma: 541	Non atopic control: 752	χ^2 ^test (allelic)	3

Large-scale genotyping using 82,935 randomly selected gene-based SNPs was carried out using the high-throughput multiplex PCR-Invader assay method as described previously [11]. The population frequency of asthma in Japan was 0.065 [12], and the statistical power of the 1^st ^stage analysis was 0.93 and 0.44 at the alpha level of 0.001 and 0.000001, respectively if the relative risk for asthma in those persons carrying a putative risk allele is 2 and the high risk allele frequency is 0.3 compared with that in persons without the allele. Therefore, our sample size may not be enough to detect a low risk allele.

The SNPs genotyped in the 1^st ^stage analysis were those identified in the JSNP project [11]. There are 2 approaches to the construction of SNP databases: one is genome-wide screening, and the other is gene-based screening. Although SNPs around genes are likely to be functional SNPs, it should be noted that SNPs outside genes have also been found to be associated with diseases.

In the present study, the distribution of allelic frequencies was largely even, with an average minor allele frequency of 24%. The SNPs with problematic genotyping in the 1^st ^stage were flagged, and we excluded these SNPs from the analysis (n = 4683). Moreover, Hardy-Weinberg equilibrium was calculated using the χ^2 ^test with 2 degrees of freedom on the basis of the observed and expected genotype frequencies; SNPs with *P *< 0.001 (n = 2160) were excluded from the analysis. After the exclusions, 76,092 autosomal SNPs were available for analysis. The significance of the differences in the allele frequencies in case-control comparisons was determined by the χ^2 ^test with 1 degree of freedom. The distribution of the observed *P *values was as follows: *P *< 0.0001, 20 SNPs (0.0263%); *P *< 0.001, 146 SNPs (0.192%); and *P *< 0.01, 1111 SNPs (1.46%). The genomic inflation factor of the study population was calculated using the method described by Devlin et al [13] and was found to be 1.13. A previous study has reported that the inclusion of different proportions of individuals from different regions of Japan in case and control groups can lead to an exaggerated number of false-positive results when the sample sizes are large, and it has recommended the exclusion of subjects belonging to the Ryukyu (southern island of Japan) cluster [14]. The patients of the present study were from the Kanto and Kinki regions, and the controls were from the Kinki region alone. On the basis of the results of a previous simulation study, we can state that the subjects from the Kanto region are not genetically different from those from the Kinki region [14]; moreover, we did not include cases or controls from the Ryukyu island. However, we cannot exclude the possibility that population stratification exists in our case-control samples.

We used a cut-off *P *value of 0.002 (corrected *P *= 0.0036) and minor allele frequency of 0.2 for allelic association for the 1^st ^stage analysis. There were 262 SNPs with *P *values < 0.002, and among them, 138 SNPs had minor allele frequencies > 0.2. We further chose 125 SNPs that were not in tightly linked with other SNPs for the 2^nd ^stage analysis. SNP typing for 2^nd ^and 3^rd ^stages was performed using the TaqMan Assay-on-Demand™ and Assay-by-Design SNP Assay Systems (Applied Biosystems, Foster City, CA) as per the manufacture's instructions. The pedigree disequilibrium test (PDT) [15] for the family-based association study (2^nd ^stage) was performed using the UNPHASED program version 2.404 . The PDT can use data from related nuclear families from extended pedigrees with multiple offspring and is valid even when there is population substructure. Eight SNPs (rs1045487, rs2288601, rs3773265, rs2273188, rs2041125, rs3213733, rs3771140, and rs2820421) were observed to be associated with asthma at the significance levels of *P *< 0.05, however, the risk alleles for asthma in 5 SNPs (rs1045487, rs2288601, rs3773265, rs2273188, and rs2041125) differed from the ones observed in the 1^st ^stage analysis. Therefore, we further genotyped the remaining 3 SNPs in a larger replication panel comprising children with atopic asthma and healthy adult controls without atopic disease (3^rd ^stage). The results of these 3 SNPs are shown in Table 3. Although the SNPs located in the 5' region of peroxisome biogenesis factor 19 (*PEX19*, rs2820421) were significantly associated with *P *< 0.05 through the 1^st ^to the 3^rd ^stage analyses, the *P *values did not reach statistically significant levels (combined, *P *= 3.8 × 10^-5^, calculated by the method described by Kirov et al [16]; statistically significant levels with Bonferroni correction, *P *= 6.57 × 10^-7^).

**Table 3:** Association results of three SNPs

7			1st stage			2nd stage	3rd stage		
Gene	rs number	Allele	Case*	Control*	*P *value (Corrected P)	PDT *P *value	Case*	Control*	*P *value

*HPCAL1*	rs3771140	A	0.84	0.77	0.0011 (0.0021)	0.0258	0.78	0.78	0.86
*PEX19*	rs2820421	A	0.55	0.48	0.0013 (0.0025)	0.045	0.53	0.47	0.0306
*IL18R1*	rs3213733	G	0.89	0.84	0.0019 (0.0035)	0.0246	0.83	0.82	0.62

*Allele frequencies

Our results revealed a few candidate genes of pediatric asthma. Though the *P *values did not reach statistically significant levels, SNPs in the 5' region of *PEX19 *were consistently associated with asthma in the 1^st ^to the 3^rd ^stage analyses. On the contrary, SNPs on hippocalcin-like 1 (*HPCAL1*, rs3771140) and on interleukin (*IL*)*18R1 *(rs3213733) were associated with asthma in the 1^st ^and 2^nd ^stage analyses, but the associations were not observed in the 3^rd ^stage analysis.

**Figure 1:**
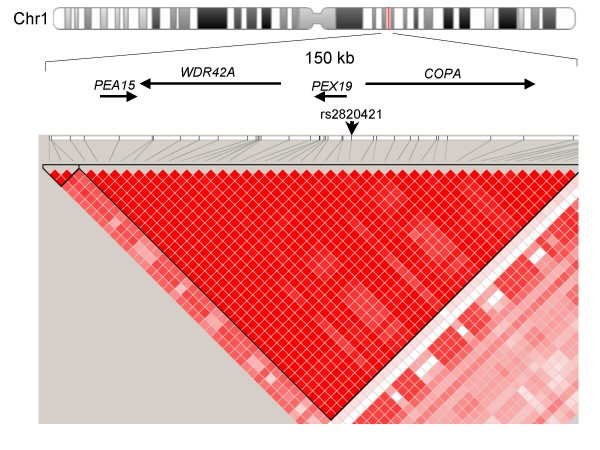
**LD map around rs2820421**. Top, locations of the genes in the 150 kb LD region. Bottom, Haploview plot of LD blocks. Red color indicates strong LD, expressed as r^2^. SNPs with an allele frequency > 0.2 are shown. An arrow indicates the location of rs2820421.

Pairwise linkage disequilibrium (LD) plots using HapMap data of the Japanese and Chinese population revealed that the SNPs were located in a tight LD region, spanning approximately 150-kb between rs822450 and rs6668576, and 4 genes (*PEA15, WDR42A, PEX19*, and *COPA*) were located in the LD region (Figure 1).

*PEX19 *is a human ortholog of the *Saccharomyces cerevisiae *gene, Pex19p, which encodes an oleic acid-inducible, farnesylated protein essential for peroxisome biogenesis [17]. Peroxisomes function to rid cells of toxic substances, such as hydrogen peroxide, or other metabolites and are essential for human survival. It has been reported that, in mice cells, Pex19p interacts with p19ARF in the cell cytoplasm and excludes p19ARF from the nucleus, leading to a concurrent inactivation of p53 function [18]. p19ARF is encoded by the cyclin-dependent kinase inhibitor 2a (Cdkn2a), and the human ortholog of Cdkn2a (CDKN2A) has been extensively examined in relation to cancer and aging [19]. Down regulation of Pex19p by its antisense expression resulted in increased levels of p19ARF, increased p53 function, and a p53/p21WAF1-mediated senescence [18]. p19ARF proteins regulate p53 pathways, and the disruption of these proteins results in aberrant cell cycle regulation and perturbation of apoptotic response [20]. Recently, it has been shown that p19Arf overexpression resulted in impaired transition from CD4(-)CD8(-) (double negative stage) to CD4(+)CD8(+) (double positive stage), leading to impaired thymocyte expansion and development [21]. Functions of other 3 genes, *WDR42A*, *PEA15 *and *COPA *for the immune systems are currently not well understood.

IL-18 was initially identified as a potent interferon gamma (IFNγ)-inducing factor, and was later shown to have the potential to induce IL-4 production. Therefore, IL-18 can induce both IFNγ and IL-4 responses depending on its cytokine environment [22]. Recently, polymorphisms in *IL18 *receptor 1 (*IL18R1*) have been reported to be associated with asthma and bronchial hyperresponsiveness in the European population [23,24]. Therefore, *IL18R1 *is a good candidate for asthma, and further replication studies are required to determine the causal variants.

In the present study, we did not detect statistically significant associations of asthma with SNPs. This may be because of the limited statistical power, considering the sample size and extent of multiple testing. Our statistical power in the 3rd-stage analysis was 95%, but the powers of the 1st- and 2nd-stage analyses were 0.95 and 0.85 with a genotypic relative risk of 2.0, and 0.41 and 0.45 with a genotypic relative risk of 1.5, respectively. Another reason is population stratification in the 1st-stage analysis. Although we collected the case and control samples from geographic regions wherein people are considered to be genetically similar, population stratification may exists in our case-control samples, leading to the inflated test statistics.

In summary, we performed a large scale genotyping study to identify the susceptibility genes for pediatric asthma. Although no SNPs attained genome-wide significance, we identified several loci with a possible association with asthma. Further studies are required to validate these results for the prevention and treatment of asthma.

## Competing interests

The authors declare that they have no competing interests.

## Authors' contributions

YI, MF, KH, and TH carried out molecular genetic study. YS, HS, KM, AA, TK, SY, ME, and MS prepared the samples and participated in the study design and coordination. TA, MT and EM participated in the design of the study, performed the statistical analysis and prepared the manuscript. All authors read and approved the final manuscript.
